# SKI-II reverses the chemoresistance of SGC7901/DDP gastric cancer cells

**DOI:** 10.3892/ol.2014.2083

**Published:** 2014-04-25

**Authors:** YING LIU, ZUAN ZHU, HONGXING CAI, QINGHUA LIU, HONGLIAN ZHOU, ZHENGQIU ZHU

**Affiliations:** 1 Department of Pathology, Xuzhou Medical College, Xuzhou, Jiangsu 221002, P.R. China; 2 Department of Gastroenterology, Affiliated Hospital of Xuzhou Medical College, Xuzhou, Jiangsu 221002, P.R. China; 3 Department of Forensic Medicine, Xuzhou Medical College, Xuzhou, Jiangsu 221002, P.R. China; 4 Department of Medical Oncology, Affiliated Hospital of Xuzhou Medical College, Xuzhou, Jiangsu 221002, P.R. China

**Keywords:** 4-[4-(4-chloro-phenyl)-thiazol-2-ylamino]-phenol, sphingosine kinase 1, glutathione, glutathione S-transferase, mitogen-activated protein kinase pathway, SGC7901/DDP cells

## Abstract

Cisplatin is frequently used in treating gastric cancers; however, acquired resistance to the drug often reduces the efficacy of therapy. The present study analyzed the efficacy of the combination of 4-[4-(4-chloro-phenyl)-thiazol-2-ylamino]-phenol (SKI-II) and cisplatin [cis-diamminedichloroplatinum (II); DDP] on the gastric cancer SGC7901/DDP cell line. The results revealed that SKI-II and DDP had a clear synergistic effect. Glutathione (GSH) and glutathione S-transferase (GST) levels decreased significantly subsequent to the cells being treated with the combination of DDP and SKI-II compared with the cells that were treated with DDP or SKI-II alone. Phosphorylated extracellular-signal-regulated kinase (p-ERK) and phosphorylated c-Jun N-terminal kinase (p-JNK) expression levels also decreased following treatment with SKI-II. The results suggested that SKI-II is able to reverse the drug resistance in human gastric carcinoma cells and enhance the antitumor effect of DDP through the ras/mitogen-activated protein kinase (MAPK) proliferation pathway.

## Introduction

Cisplatin [cis-diamminedichloroplatinum (II); DDP] is a platinum-based drug that is frequently used in the treatment of various cancers, including non-Hodgkin’s lymphoma and breast, testicular, ovarian, head and neck, esophageal and bladder cancer (1–3). Despite its therapeutic use, the intrinsic resistance that is acquired following continuous treatment limits the benefit of cisplatin in cancer therapy. Therefore, the identification of new anticancer drugs is required to optimize the performance of DDP. Sphingosine-1-phosphate (S1P) is a bioactive lipid that has been implicated in regulating a variety of cell biological responses, including cell proliferation, survival, differentiation and migration (4). There is a great deal of interest in the roles that S1P and other sphingolipids play in cell signaling and the response to stress (5–8). Furthermore, numerous studies have linked S1P to various aspects of cell motility, chemotaxis and metastasis in mammalian and SGC7901/DDP cells (9–11).

The enzyme sphingosine kinase (SphK) is an oncogene that is tightly regulated by a number of growth factors and protein kinases. SphK is able to increase the level of intracellular S1P. 4-[4-(4-chloro-phenyl)-thiazol-2-ylamino]-phenol (SKI-II) is mainly used to inhibit SphK activity. SKI-II is able to inhibit S1P formation in cells that express drug transport proteins, including P-glycoprotein (P-gp), multidrug resistance (MDR)-associated protein-1 (MRP1) and glutathione S-transferase (GST). The majority of antitumor activities correlate with the concentrations of the proteins that are present in the blood and tumors (12). The results provide further validation for SphK as a cancer therapeutic target, as well as small molecule inhibitors of SphK. The present study analyzed the effects and explored the mechanisms of the combination of SKI-II and DDP in SGC7901/DDP cells, which may aid the further clinical application of this combination in chemotherapy.

## Materials and methods

### Reagents

The following reagents and detection kits were used: SKI-II (Sigma-Aldrich Co., St Louis, MO, USA). SKI-II dissolved in dimethyl sulfoxide (DMSO); RMPI-1640 medium (Beyotime Institute of Biotechnology, Haimen, Jiangsu, China); fetal bovine serum (FBS; Tianjin Hao Yang Biological Manufacture Co., Ltd., Tianjin, China); 3-(4, 5-dimethylthazol-2-yl)-2, 5-diphenyl tetrazolium bromide (MTT; Amresco, USA); glutathione (GSH); a GST detection kit (Nanjing Jiancheng Bioengineering Institute, Nanjing, Jiangsu, China) and a total RNA Extraction kit (Sangong Biotechnology, Shanghai, China).

Monoclonal antibodies (mAb) against P-gp, MRP1, phosphorylated extracellular-signal-regulated kinase (p-ERK) and phosphorylated c-Jun N-terminal kinase (p-JNK; Santa Cruz Biotechnology Institute, Shanghai, China) were also used.

### Cell line and cell culture

The human gastric carcinoma SGC7901/DDP cell line was purchased from the Nanjing KeyGen Biological Co., Ltd. (Nanjing, Jiangsu, China). The cells were cultured in RMPI-1640 medium (pH 7.2–7.4) containing 100 ml/l fetal calf serum (FCS) and 10 ml/l penicillin and streptomycin at 37°C in a humidified (95%) incubator containing 95% air and 50 ml/l 5% CO_2_. The SGC7901/DDP cells were cultured for 24 h and then incubated with various concentrations of SKI-II (μmol/l) + DDP (mg/l) for 48 h.

### Cytotoxicity and MDR reversal assay

The cells were seeded into 96-well plates at 2×10^4^ cells/well. Various concentrations of the compound SKI-II (μmol/l) + DDP (mg/l; 0+0, 0+0.625, 0+1.25, 0+2.5, 0+5, 1.25+0, 1.25+0.625, 1.25+1.25, 1.25+2.5 and 1.25+5) were subsequently added and incubated for 48 h. The IC_50_ value of DDP in the absence or presence of 1.25 μmol/l SKI-II for 48 h was calculated from the plotted results using the untreated cells as 100% by probit analysis using SPSS 16.0 (SPSS, Inc., Chicago, IL, USA). The reversion fold (RF) values, as the potency of reversal, were calculated using the formula RF = IC_50_ of DDP alone/IC_50_ of DDP incubated with the test compounds (8,10). Triplicate experiments with triplicate samples were performed.

### Immunocytochemistry assay

The exponentially-growing cells were cultured in a 24-well plate at a density of 1×10^4^ cells/well. Various concentrations of the SKI-II (μmol/l) + DDP compound (mg/l; 0+0, 0+2.5, 1.25+0, 1.25+2.5, 5+0, 5+2.5, 10+0 and 10+2.5) were subsequently added and incubated for 48 h. An IHC detection reagent and DAB kit (Santa Cruz Biotechnology Institute) were used to examine the expression of P-gp, MRP1, p-JNK and p-ERK. The treated cells were rinsed three times with 0.01 M phosphate-buffered solution (PBS) and fixed for 30 min with 4% paraformaldehyde at 4°C. Following this, the cells were incubated with 0.3% TritonX-100 for 20 min and 3% H_2_O_2_ for 10 min. The cells were then blocked with 10% normal goat serum for 1 h at room temperature. Primary antibodies were added and incubated at 4°C overnight. Subsequent to the cells being rinsed three times with cold PBS, the secondary antibody was added and incubated at room temperature for 30 min. Finally, the cells were rinsed with 0.01 M PBS three times and incubated with the DAB complexes for 20 min. The cells were observed and images were captured using a phase contrast microscope (Olympus DP-11, Tokyo, Japan). Triplicate experiments with triplicate samples were performed.

### Western blot analysis

The cells were plated on culture flasks at a density of 1×10^4^ cells/ml with various concentrations of SKI-II (μmol/l) + DDP (mg/l; 0+0, 0+2.5, 1.25+0, 1.25+2.5, 5+0, 5+2.5, 10+0 and 10+2.5) for 48 h. Following each experiment, the cells were rinsed with 0.01 mol/l PBS three times and 200 μl lysate containing 150 ml/l protease inhibitor was added, followed by use of an ice bath for 15 min. The cells were then scraped from the flasks, harvested and centrifuged at 15,000 × g for 20 min for supernatant collection of the total proteins. The protein contents were determined by a Lowry assay. Sodium dodecyl sulfate polyacrylamide gel electrophoresis (SDS-PAGE) gel sample buffer was added to the lysates, which were heated to 100°C for 5 min. Following this, 100 μg protein was loaded into each well of a 12.5% SDS-PAGE gel for electrophoresis and the targeted protein was transferred onto a nitrocellulose membrane. The membrane was blocked using 5% skimmed milk for 2 h at room temperature, incubated overnight at 4°C with primary antibodies directed against P-gp, MRP1, p-ERK, p-JNK (all diluted 1:500 in primary antibody dilution buffer) and β-actin (loading control, diluted 1:1000 in primary antibody dilution buffer). The membrane was washed three times for 5 min each in washing buffer and incubated with the appropriate AP-conjugated secondary antibody (rabbit anti-P-gp, MRP1, p-JNK, p-ERK and rabbit anti-β-actin diluted 1:1000 in secondary antibody dilution buffer) for 2 h at room temperature. The blotted protein bands were visualized using the BCIP/NBT Alkaline Phosphatase Color Development kit (Beijing Biotech Co., Ltd., Beijing, China). The developed films were digitized using an Epson Perfection 2480 scanner (Seiko Corp, Nagano, Japan). The optical density (OD) values of the proteins were obtained using Glyko Bandscan software (Glyko, Novato, CA, USA).

### Intracellular GSH and GST quantification

Subsequent to being incubated with various concentrations of SKI-II (μmol/l) + DDP (mg/l; 0+0, 0+2.5, 1.25+0, 1.25+2.5, 5+0, 5+2.5, 10+0 and 10+2.5) for 48 h, 1×10^5^ cells were collected and homogenized with 10 mM HCl. The homogenate was placed in a 96-well microplate and measured using a spectrophotometer (Shimadzu UV-1208; Shimadzu, Kyoto, Japan) at 412 nm. The reactions between 5,5-dithiobis(2-nitrobenzoic acid) (DNTB) and intracellular GSH and GST were quantified by comparing the absorbance at 412 nm for each sample with that of a GSH standard solution (0–100 μM/l). The absorbance was measured 10 min after the addition of DNTB.

### RNA extraction and semi-quantitative polymerase chain reaction (PCR)

The cells were plated on culture flasks at a density of 1×10^4^ cells/ml with various concentrations of SKI-II (μmol/l) + DDP (mg/l; 0+0, 0+2.5, 1.25+0, 1.25+2.5, 5+0, 5+2.5, 10+0 and 10+2.5) for 48 h. Total RNA was isolated using a Total RNA Extraction kit (Tiangen Biotech Co., Ltd., Beijing, China), and DNase treatment was performed using the RNase-Free DNase Set according to the manufacturer’s instructions. Reverse transcription of ≥10 μg total RNA was performed in a total volume of 100 μl containing 250 pmol oligo (dT)18, 80 U rRNasin ribonuclease inhibitor and 500 U Moloney murine leukemia virus reverse transcriptase in 50 mM Tris-HCl (pH 8.3), 75 mM KCl, 3 mM MgCl_2_, 10 mM DTT and 0.5 mM dNTPs. The total RNA and oligo (dT)18 solutions were initially heated at 70°C for 10 min and then immediately chilled on ice. The other reagents were added and incubated for 15 min at 30°C and then 60 min at 42°C.

The primers and TaqMan probes for MRP1 and GST were designed using Primer Express software (Tiangen Biotech Co., Ltd.). The primers and TaqMan probes for β-actin were purchased from Tiangen Biotech Co., Ltd.. The evaluation of β-actin expression, used as a control of the RNA amount, was performed using the following primer sequences: forward, 5′-GTGGGGCGCCCCAGGCACCA-3′ and reverse, 5′-CTC CTTAATGTCACGCACGATTT-3′, which yielded a 500-bp product. The other primers sequences that were used were: MRP1 forward, 5′-CCCCATGAATCCAAGATACCTA-3′ and reverse, 5′-CCTTACCATTTGGAGATGAAGC, which yielded a 1808-bp product; GST sequence forward, 5′-ACC TTCTTTGGTGGAACCTGTA-3′ and reverse, 5′-AAAGGC ATTAGGGTTGTTCTGA-3′, which yielded a 1016-bp product.

Quantification of the target cDNA (MRP1 and GST) and an internal reference gene (β-actin) was conducted using a fluorescence-based quantitative PCR method. PCR was performed in a 25-μl reaction volume containing cDNA equivalent to 1–10 ng total RNA and 200 nM of each primer, 100 nM of probe and 12.5 U TaqMan universal PCR Master Mix (containing 1XTaqMan buffer, 200 μM dATP, dCTP and dGTP and 400 μM dUTP, 5 mM MgCl_2_, 1.25 U AmpliTaqGold and 0.5 U AmpErase UNG). The thermal cycling conditions were 50°C for 2 min and 95°C for 10 min, followed by 45 cycles at 95°C for 15 sec and 60°C for 1 min. The relative quantification of gene expression was performed using the relative standard curve method. The standard curve was created automatically using the ABI PRISM 7700 Detection System software (Applied Biosystems, Foster City, CA, USA) by plotting the threshold cycle (CT) against each input amount of the control total RNA (16, 4, 1, 0.25, 0.063, 0.016 and 0.0039 ng total starting RNA), prepared from the SGC7901/DDP human gastric carcinoma cells. The coefficient of linear regression for each standard curve was >0.990. For each unknown sample, the relative amount was calculated using a linear regression analysis from the respective standard curve. A relative target gene expression value was obtained by dividing the target gene value by the β-actin value (internal reference gene).

### Experimental animals and tumor-bearing nude mouse model

A total of 24 female BALB/C nude mice, aged 4 weeks and weighing 15–19 g, were obtained from the Experimental Animal Center of Xuzhou Medical College. All experiments were carried out in accordance with the National Institutes of Health Guide for the Care and Use of Laboratory Animals. This study was approved by the ethics committee of XuZhou Medical College.

Human gastric cancer SGC7901/DDP cells were implanted under the skin on the right side in 16 BALB/C nude mice. The inoculation volume was 0.1 ml (1×10^7^ living cells/ml). Following the inoculation, tumor nodes appeared at the inoculated site, which were observed continuously for 1 week. The tumors were formed from hard tumor nodes at the inoculated site and increasing nodal enlargement. The mice were raised in an appropriate animal house and fed with cool boiled water and fodder sterilized with steam. The wood chips that were used for the bedding were sterilized with steam and the mouse cages were sterilized with a disinfectant solution. The water, fodder, wood chips and cages were changed and cleaned once a day, and the ambient temperature and humidity were kept at 22±1°C and 60±10%, respectively, on a 12-h day/night cycle. The maximum tumor diameter was measured using a vernier caliper.

All the animal experiments were performed in compliance with the national laws relating to animal research. The 16 BALB/C tumor-bearing nude mice were randomized into four groups, with four mice in each group. The normal group consisted of mice that were treated with nothing. The DDP mouse group were treated with a 2.5 mg/kg intraperitoneal perfusion (i.p.) of DDP for 48 h. The SKI-II mouse group was treated with 0.3 mg/kg i.p. SKI-II for 48 h and the DDP + SKI-II mouse group were treated with 0.3 mg/kg i.p. SKI-II and 2.5 mg/kg i.p. DDP for 48 h. The mice were then sacrificed by cervical dislocation at 48 h subsequent to i.p. administration. The tumors were removed to measure their weight. Next, certain tumors were mounted on glass slides for immunohistochemical (IHC) staining. Other tumors were collected and stored at −80°C for western blotting and GSH and GST quantification.

### Immunohistochemical staining

The IHC detection reagent and DAB kit were used to examine the expression of P-gp, MRP1, p-JNK and p-ERK. Briefly, the deparaffinized sections were heated in a 700 W microwave oven for 12 min to retrieve the antigens and then cooled to room temperature. Endogenous peroxide activity was blocked using 3% H_2_O_2_ for 10 min. The sections were washed with 0.01 M PBS, blocked with 10% rabbit serum for 15 min to reduce non-specific antibody binding and incubated with the respective primary antibody (against P-gp, MRP1, p-ERK or p-JNK) at 4°C overnight. The other steps were similar to those described in the IHC assay.

### Western blotting

The tumors were collected over ice and mechanically lysed in RIPA Lysis Buffer (P0013B; Beyotime Institute of Biotechnology), which contained 50 mM Tris (pH 7.4), 150 mM NaCl, 1% Triton X-100, 1% sodium deoxycholate, 0.1% SDS, sodium orthovanadate and 1 mM phenylmethylsulphonyl fluoride (PMSF). The mucosa was homogenized for 30 sec and kept on ice for 20 min. The homogenates were centrifuged at 12,000 × g for 30 min at 4°C, and the supernatants that contained the cytoplasmic components were retained and stored at −80°C and thawed once. The nuclear pellets were dissolved in RIPA Lysis Buffer and PMSF, centrifuged at 12,000 × g for 20 min at 4°C and the supernatant extracts collected. The nuclear extracts were aliquoted and stored at −80°C for western blotting analysis of p65 protein activity. The protein concentrations were determined using the bicinchoninic acid (BCA) protein assay. Equal amounts of protein were resolved in SDS-polyacrylamide gels and transferred electrophoretically onto a nitrocellulose membrane (Amersham Biosciences, Amersham, UK). The other steps were similar to those described in the western blot analysis.

### Tumor GSH and GST quantification

To detect the activity of GST and the contents of GSH, the tumors were excised and subsequently homogenized in normal saline at 4°C. The homogenate was centrifuged at 4,000 × g for 10 min and the supernatant was retained. The homogenate was placed in a 96-well microplate and the absorbance was measured at 412 nm was using a spectrophotometer (Schimadzu UV-1208). The other steps were similar to those described in the intracellular GSH and GST quantification.

### Statistical analysis

The results are presented as the mean ± SD of three replicated experiments. A one-way ANOVA was performed to determine the differences among the groups. All the statistical analyses were performed using GraphPad Prism 5 (GraphPad Software Inc., La Jolla, CA, USA). P<0.05 was considered to indicate a statistically significant difference.

## Results

### SKI-II reverses the resistance of cells to DDP

SKI-II in combination with DDP had a greater effect on the SGC-7901/DDP cells compared with DDP or SKI-II alone (P<0.05). The resistance index of the SGC7901/DDP cells to DDP was 6.3. Following the treatment with SKI-II, the IC_50_ of DDP to the SGC7901/DDP cells was reduced from 3.33 μmol/l to 2.062 μmol/l by 1.615-fold (Table I).

### Expression of P-gp, MRP-1, p-ERK and p-JNK in immunocytochemistry assay

The immunocytochemistry assay *in vitro* confirmed the expression of P-gp, MRP-1, p-ERK and p-JNK in the human gastric carcinoma cells. The number of P-gp-, MRP-1-, p-ERK- and p-JNK-positive cells in the SKI-II (μmol/l) + DDP (mg/lU; 5+0, 10+0, 5+2.5 and 10+2.5) groups were significantly decreased (P<0.05). The immunocytochemistry assay *in vivo* confirmed the expression of P-gp, MRP-1, p-ERK and p-JNK in the tumors. The number of P-gp-, MRP-1-, p-ERK- and p-JNK-positive cells *in vitro* and *in vivo* all decreased (P<0.05) following the treatment with SKI-II alone and significantly decreased following the treatment with the DDP + SKI-II group (P<0.05; Tables II and III).

### Expression of P-gp, MRP-1, p-ERK and p-JNK in the western blot assay

**Figure 1:**
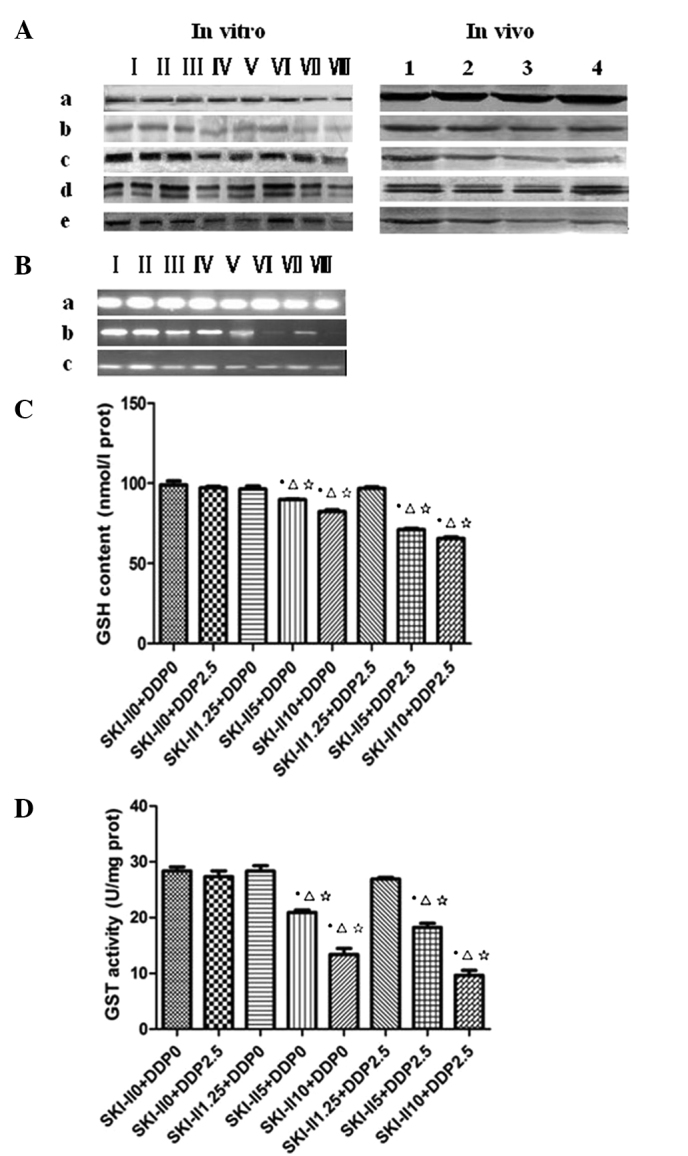
P-gp, MRP1, p-ERK and p-JNK expression was downregulated by SKI-II and DDP, as determined by western blotting. (A) a, β-actin; b, P-gp; c, MRP1; d, p-ERK; and e, p-JNK. I, control; II, DDP 2.5; III, SKI-II 1.25; IV, SKI-II 5; V, SKI-II 10; VI, DDP 2.5 + SKI-II 1.25; VII, DDP 2.5 + SKI-II 5; and VIII, DDP 2.5 + SKI-II 10. 1, Control; 2, DDP; 3, SKI-II and 4, DDP+SKI-II. (B) MRP1, GSH and GST expression was downregulated following treatment with SKI-II and DDP, as observed by RT-PCR. a, β-actin; b, GST; and c, MRP1. I, control; II, DDP 2.5; III, SKI-II 1.25; IV, SKI-II 5; V, SKI-II 10; VI, DDP 2.5 + SKI-II 1.25; VII, DDP 2.5 + SKI-II 5; and VIII, DDP 2.5 + SKI-II 10. The effects of SKI-II+DDP on (C) GSH contents and (D) GST activity are shown following the treatment. ^*^P<0.05 vs. normal group; ^Δ^P<0.05 vs. DDP 2.5 group; ^⋆^P<0.05 vs. SKI-II1.25 group. P-gp, P-glycoprotein; MRP1, multidrug resistance-associated protein-1; p-ERK, phosphorylated extracellular-signal-regulated kinase; p-JNK, phosphorylated c-Jun N-terminal kinase; SKI-II, 4-[4-(4-chloro-phenyl)-thiazol-2-ylamino]-phenol; DDP, cis-diamminedichloroplatinum (II); GSH, glutathione; GST, glutathione S-transferase.

The result of the western blot assay *in vitro* demonstrated that P-gp, MRP-1, p-ERK and p-JNK were expressed in the human gastric carcinoma cells. Compared with the DDP (2.5 mg/l) group, the expression of P-gp, MRP-1, p-ERK and p-JNK decreased in the SKI-II (μmol/l) + DDP (mg/l; 5+0, 10+0, 5+2.5 and 0+2.5; P<0.05; Table IV). The result of the western blot assay *in vivo* demonstrated that P-gp, MRP-1, p-ERK and p-JNK were expressed in the tumors. Compared with the DDP group, the expression of P-gp, MRP-1, p-ERK and p-JNK decreased (P<0.05) in the tumors following the treatment with SKI-II alone and significantly decreased following the treatment with the DDP + SKI-II group (P<0.05; Fig. 1A, Table V).

### Expression levels of MRP-1, GST messenger RNA (mRNA) in SGC7901/DDP cells

The expression levels of MRP-1 mRNA in the SGC7901/DDP cells were calculated as: (Ratio of the amount of MRP-1 mRNA/amount of β-actin mRNA in the DDP-treated cells)/(ratio of the amount of MRP1 mRNA/amount of β-actin mRNA in cells at 48 h following SKI-II and/or DDP treatment). The MRP-1 levels peaked in the SKI-II (μmol/l) + DDP (mg/l; 0+0;0+2.5) groups, but had decreased in the SKI-II (μmol/l) + DDP (mg/l; 5+0; 10+0, 5+2.5 and 10+2.5) treatment groups after 48 h. In the SKI-II (μmol/l) + DDP (mg/l; 5+0, 10+0, 5+2.5 and 10+2.5)-treated cells, the combination of the two drugs resulted in a significantly lower level of MRP1 expression compared with the cells that were treated with SKI-II alone at the 48-h time-point (P<0.05). The expression of GST mRNA in the SGC7901/DDP cells was decreased by SKI-II + DDP (mg/l; 5+2.5 and 10+2.5) treatment after 48 h, exhibiting a significant suppression of GST expression compared with SKI-II or DDP treatment alone (P<0.05; Fig. 1B). This suggested that SKI-II treatment enhanced DDP toxicity against the SGC7901/DDP cells through the suppression of GST expression, which is normally enhanced by DDP exposure in the SGC7901/DDP cells.

### Contents of GSH and GST

*In vitro*, the expression of GSH decreased significantly (P<0.05) in the cells subsequent to being treated with SKI-II alone (5 μmol/l and 10 μmol/l), and significantly decreased subsequent to being treated with SKI-II (μmol/l) + DDP (mg/l; 5+2.5 and 10+2.5; P<0.05). The GST activity significantly decreased in the SKI-II (μmol/l) + DDP (mg/l; 5+2.5 and 10+2.5) groups when compared with SKI-II alone (5 umol/l and 10 umol/l; P<0.05). *In vivo*, GSH decreased (P=0.027; P<0.05) in the tumors subsequent to being treated with SKI-II alone and significantly decreased following treatment with DDP + SKI-II (P<0.05; Fig. 1C and D). The GST activity significantly decreased in the DDP + SKI-II group when compared with the SKI-II alone group (P<0.05).

## Discussion

Gastric cancer is a common malignant tumor of the alimentary tract and its incidence rate is among the three leading kinds of neoplasm in various regions of China (13,14). Conventional treatments for advanced gastric cancer include extended resection, radiotherapy and chemotherapy, which have little effect on the survival rate of affected patients. Cisplatin is a platinum chemotherapeutic agent that is used in a variety of malignancies. The major limitation in the clinical application of cisplatin has been the development of cisplatin resistance by tumors. A number of experimental strategies to overcome cisplatin resistance are at the preclinical or clinical levels, including the introduction of the bax gene, the inhibition of the JNK pathway, the introduction of a functional p53 gene and the treatment of tumors with aldose reductase inhibitors (15,16). Of particular significance are the combinations of platinum drug treatments with other drugs, radiation and the emerging gene therapy regimens. The present study analyzed the effect of the combination of SKI-II and DDP on the SGC7901/DDP cells and identified that SKI-II in combination with DDP had an improved effect compared with DDP or SKI-II alone. Following the treatment with SKI-II, the IC_50_ of DDP to the SGC7901/DDP cells was reduced. Furthermore, the present study explored the mechanisms of SKI-II *in vitro* and *in vivo*.

MDR is an unfavorable factor that causes the failure of treatments against cancer cells (17). MDR occurs when cancer cells acquire a simultaneous resistance to various chemotherapeutic agents that have no structural or functional similarities (18). The mechanisms that are involved in MDR include the overexpression of mutispecific ATP-dependent drug efflux pumps, including P-gp, MRP1 and BCRP (ABCG2), which reduce the available concentration of the drug for the cancer cells (19). The present study identified that the combination of SKI-II and DDP was able to reverse the MDR of the SGC7901/DDP cells via the downregulation of P-gp and MRP1, which increased the concentrations of DDP in the SGC7901/DDP cells.

Endogenous antioxidants, including reduced GSH, GSH peroxidase (GSH-PX), superoxide dismutase (SOD) and catalase (CAT), are compounds that act as free radical scavengers. These antioxidants are electron donors and react with the free radicals to form harmless products such as water. Therefore, antioxidants protect against oxidative stress and prevent damage to cells (20). Similarly, GST is a soluble protein that is located in the cytosol and plays a significant role in cell protection. The present study identified that the combination of SKI-II and DDP decreased the levels of GSH and GST in the SGC7901/DDP cells, which decreased the protection possibility of GSH and GST to the cells. Therefore, the inhibition rate of the SGC7901/DDP cells that were treated with SKI-II and DDP increased significantly *in vitro* and *in vivo*.

MAPKs belong to a large family of proline-directed serine-threonine protein kinases that are significant signaling components in the conversion of extracellular signals into an intracellular response through a series of phosphorylation cascades (21). The ultimate effects of MAPK activation and phosphorylation depend on the ability of the kinases to induce the appropriate gene expression events. Three MAP kinase pathways, ERK, JNK and p38, have been identified and well studied (22,23). The expression and activation of the JNK and ERK MAPK pathway correlate with prognosis and affects the therapeutic outcome in several types of cancer (24,25). The present study revealed that the combination of SKI-II and DDP may decrease the MAPK pathway by decreasing the expression of ERK and JNK in the SGC7901/DDP cells. Wasserman *et al* identified that the activation of MAPK also induced the mRNA expression of immediate early genes, including c-jun and c-fos, and transcriptionally activated the antioxidant/electrophile response element (ARE/EpRE) and the chloramphenicol acetyltransferase reporter gene, which are present in a number of stress-response genes, including GST, quinone reductase and heme oxygenase 1 (26–29). Furthermore, the present study revealed that the combination of SKI-II and DDP may also decrease the levels of GSH and GST in the SGC7901/DDP cells. This emphasizes the complexity of MAPK signaling.

In summary, the results of the present study have shown that SKI-II is able to reverse the chemoresistance of SGC7901/DDP gastric cancer cells by decreasing the levels of P-gp and MRP-1, which increases the concentrations of DDP in the SGC7901/DDP cells. Furthermore, SKI-II decreased the levels of GSH and GST in the cells, which decreased the protection possibility of GSH and GST to the SGC7901/DDP cells. The present study demonstrated that the combination of SKI-II and DDP was able to regulate the MAPK pathways by decreasing the expression of ERK and JNK in the SGC7901/DDP cells. The study has provided significant insights into the signal transduction pathways that are induced by the combination of SKI-II and DDP, which activate MAPK pathways, leading to a decrease in GST for cellular protection signaling. Therefore, further understanding of the regulatory system of MDR, GSH and MAPK signaling is required for the application of SKI-II and DDP in clinical settings.

## Figures and Tables

**Table I:** Reversing effect of SKI-II on SGC7901/DDP cells.

Group, SKI-II (μmol/l) + DDP (mg/l)	Inhibition rate (%)	IC_50_	RF
Pre-reversion			
0+0	0.00±0.000		1.615
0+0.625	0.18±0.008		
0+1.25	0.40±0.011	3.330	
0+2.5	1.75±0.063		
0+5	4.08±0.024		
Post-reversion			
1.25+0	0.00±0.000		
1.25+0.625	1.48±0.013		
1.25+1.25	3.50±0.320		
1.25+2.5	4.28±0.042	2.062	
1.25+5	12.15±0.175		

Data are presented as the mean ± SD; n=6. SKI-II, 4-[4-(4-chloro-phenyl)-thiazol-2-ylamino]-phenol; DDP, cis-diamminedichloroplatinum (II); RF, reversion fold.

**Table II:** Expression of P-gp, MRP-1, p-ERK and p-JNK in the immunocytochemistry assay *in vitro*.

Groups, SKI-II (μmol/l) + DDP (mg/l)	P-gp	MRP1	p-JNK	p-ERK
0+0	70.90±0.77	71.47±0.47	68.47±0.52	70.50±0.50
0+2.5	71.06±1.20	72.55±0.58	67.95±0.90	71.53±0.54
0+1.25	71.51±0.48	71.28±0.77	68.57±0.39	70.43±0.65
0+5	54.01±1.02^a^^b^^c^	53.37±0.61^a^^b^^c^	54.60±1.00^a^^b^^c^	52.86±1.00^a^^b^^c^
0+10	34.13±0.99^a^^b^^c^	39.55±0.50^a^^b^^c^	40.44±0.45^a^^b^^c^	39.73±0.36^a^^b^^c^
1.25+2.5	71.64±0.70	69.43±0.65	68.12±0.35	69.47±0.63
5+2.5	40.20±1.36^a^^b^^c^	44.31±0.90^a^^b^^c^	45.73±+0.26^a^^b^^c^	44.61±0.53^a^^b^^c^
10+2.5	28.64±0.85^a^^b^^c^	29.39±1.05^a^^b^^c^	30.57±0.70^a^^b^^c^	30.63±0.71^a^^b^^c^

a P<0.05 vs. control group,

b P<0.05 vs. DDP 2.5 group and

c P<0.05 vs. SKI-II 1.25 group.

Data are presented as the mean ± SD; n=3. P-gp, P-glycoprotein; MRP1, multidrug resistance (MDR)-associated protein-1; SKI-II, 4-[4-(4-chloro-phenyl)-thiazol-2-ylamino]-phenol; DDP, cis-diamminedichloroplatinum (II); p-ERK, phosphorylated extracellular-signal-regulated kinase; p-JNK, phosphorylated c-Jun N-terminal kinase.

**Table III:** Expression of P-gp, MRP-1, p-ERK and p-JNK in immunocytochemistry assay *in vivo*.

	Protein expression rate (%)			
	
Group	P-gp	MRP1	p-ERK	p-JNK
Control	72.313±1.560	73.522±1.559	72.783±0.916	74.800±0.699
DDP	70.546±0.602	69.323±0.472	71.867±0.787	73.003±0.284
SKI-II	51.503±0.662^a^^b^	50.624±0.673^a^^b^	52.500±0.545^a^^b^	52.173±0.982^a^^b^
DDP+SKI-II	45.637±0.372^a^^b^^c^	42.321±0.403^a^^b^^c^	44.534±0.561^a^^b^^c^	46.040±0.830^a^^b^^c^

a P<0.05 vs. control,

b P<0.05 vs. DDP group and

c P<0.05 vs. SKI-II group.

Data are presented as the mean ± SD; n=4. P-gp, P-glycoprotein; MRP1, multidrug resistance (MDR)-associated protein-1; SKI-II, 4-[4-(4-chloro-phenyl)-thiazol-2-ylamino]-phenol; DDP, cis-diamminedichloroplatinum (II); p-ERK, phosphorylated extracellular-signal-regulated kinase; p-JNK, phosphorylated c-Jun N-terminal kinase.

**Table IV:** OD value of P-gp, MRP1, p-JNK and p-ERK in all the groups *in vivo* in the western blot assay.

	OD			
	
Group	P-gp	MRP1	p-JNK	p-ERK
Control	1.250±0.002	1.231±0.002	1.044±0.035	1.089±0.004
DDP 2.5	1.244±0.021	1.222±0.001	1.002±0.008	1.078±0.005
SKI-II 1.25	1.231±0.004	1.224±0.003	1.009±0.009	1.086±0.004
SKI-II 5	0.838±0.007^a^^b^^c^	0.844±0.004^a^^b^^c^	0.867±0.003^a^^b^^c^	0.864±0.00^a^^b^^c^
SKI-II 10	0.744±0.005^a^^b^^c^	0.722±0.005^a^^b^^c^	0.728±0.002^a^^b^^c^	0.735±0.03^a^^b^^c^
DDP 2.5+SKI-II 1.25	1.225±0.005	1.222±0.002	1.004±0.14	1.074±0.007
DDP 2.5+SKI-II 5	0.695±0.005^a^^b^^c^	0.706±0.004^a^^b^^c^	0.745±0.006^a^^b^^c^	0.747±0.01^a^^b^^c^
DDP 2.5+SKI-II 10	0.644±0.004^a^^b^^c^	0.669±0.003^a^^b^^c^	0.651±0.004^a^^b^^c^	0.668±0.00^a^^b^^c^

a P<0.05 vs. control,

b P<0.05 vs. DDP 2.5 group and

c P<0.05 vs. SKI-II 1.25 group.

Data are presented as the mean ± SD; n=3. OD, optical density; P-gp, P-glycoprotein; MRP1, multidrug resistance (MDR)-associated protein-1; SKI-II, 4-[4-(4-chloro-phenyl)-thiazol-2-ylamino]-phenol; DDP, cis-diamminedichloroplatinum (II); p-ERK, phosphorylated extracellular-signal-regulated kinase; p-JNK, phosphorylated c-Jun N-terminal kinase.

**Table V:** OD value of P-gp, MRP1, p-JNK and p-ERK in all the groups *in vitro* in the western blot assay.

	OD			
	
Group	P-gp	MRP1	p-ERK	p-JNK
Control	1.313±0.018	1.220±0.0265	1.093±0.203	1.083±0.040
DDP	1.300±0.017	1.220±0.0252	1.060±0.044	1.023±0.054
SKI-II	1.047±0.041^a^^b^	0.997±0.068^a^^b^	0.813±0.015^a^^b^	0.817±0.026^a^^b^
DDP+SKI-II	0.870±0.056^a^^b^^c^	0.810±0.026^a^^b^^c^	0.713±0.020^a^^b^^c^	0.680±0.023^a^^b^^c^

a P<0.05 vs. control,

b P<0.05 vs. DDP group and

c P<0.05 vs. SKI-II group.

Data are presented as the mean ± SD; n=4. OD, optical density; P-gp, P-glycoprotein; MRP1, multidrug resistance (MDR)-associated protein-1; SKI-II, 4-[4-(4-chloro-phenyl)-thiazol-2-ylamino]-phenol; DDP, cis-diamminedichloroplatinum (II); p-ERK, phosphorylated extracellular-signal-regulated kinase; p-JNK, phosphorylated c-Jun N-terminal kinase.

## References

[1] Lekakis L, Tryfonopoulos D, Pistamatzian N (2012). Salvage chemotherapy with cisplatin and 5-fluorouracil in metastatic breast cancer. Particular activity against liver metastases. Anticancer Res.

[2] García-Velasco A, Durán I, García E (2012). Biological markers of cisplatin resistance in advanced testicular germ cell tumours. Clin Transl Oncol.

[3] Yanagawa M, Tatsumi M, Miyata H (2012). Evaluation of response to neoadjuvant chemotherapy for esophageal cancer: PET response criteria in solid tumors versus response evaluation criteria in solid tumors. J Nucl Med.

[4] Kumar A, Saba JD (2009). Lyase to live by: sphingosine phosphate lyase as a therapeutic target. Expert Opin Ther Targets.

[5] Hla T (2004). Physiological and pathological actions of sphingosine 1-phosphate. Semin Cell Dev Biol.

[6] Ogretmen B, Hannun YA (2004). Biologically active sphingolipids in cancer pathogenesis and treatment. Nat Rev Cancer.

[7] Spiegel S, Milstien S (2002). Sphingosine 1-phosphate, a key cell signaling molecule. J Biol Chem.

[8] Taha TA, Argraves KM, Obeid LM (2004). Sphingosine-1-phosphate receptors: receptor specificity versus functional redundancy. Biochim Biophys Acta.

[9] Kumar A, Wessels D, Daniels KJ (2004). Sphingosine-1-phosphate plays a role in the suppression of lateral pseudopod formation during Dictyostelium discoideum cell migration and chemotaxis. Cell Motil Cytoskeleton.

[10] Oskouian B, Saba JD (2004). Death and taxis: what non-mammalian models tell us about sphingosine-1-phosphate. Semin Cell Dev Biol.

[11] Spiegel S, English D, Milstien S (2002). Sphingosine 1-phosphate signaling: providing cells with a sense of direction. Trends Cell Biol.

[12] French KJ, Upson JJ, Keller SN (2006). Antitumor activity of sphingosine kinase inhibitors. J Pharmacol Exp Ther.

[13] Roukos DH, Kappas AM (2005). Perspectives in the treatment of gastric cancer. Nat Clin Pract Oncol.

[14] Menges M, Schmidt C, Lindemann W (2003). Low toxic neoadjuvant cisplatin, 5-fluorouracil and folinic acid in locally advanced gastric cancer yields high R-0 resection rate. J Cancer Res Clin Oncol.

[15] Zhao Z, Wang J, Tang J (2012). JNK-and Akt-mediated Puma expression in the apoptosis of cisplatin-resistant ovarian cancer cells. Biochem J.

[16] Hasegawa M, Ishiguro K, Ando T, Goto H (2012). Geranylgeranylacetone attenuates cisplatin-induced reductions in cell viability by suppressing the elevation of intracellular p53 content without heat shock protein induction. Nagoya J Med Sci.

[17] Vasconcelos FC, Cavalcanti GB, Silva KL (2007). Constrasting features of MDR phenotype in leukemias by using two fluorochromes: implications for clinical practice. Leuk Res.

[18] Patel NH, Rothenberg ML (1994). Multidrug resistance in cancer chemotherapy. Invest New Drugs.

[19] Legrand O, Simonin G, Beauchamp-Nicoud A (1999). Simultaneous activity of MRP1 and Pgp is correlated with in vitro resistance to daunorubicin and with in vivo resistance in adult acute myeloid leukemia. Blood.

[20] Valko M, Rhodes CJ, Moncol J (2006). Free radicals, metals and antioxidants in oxidative stress-induced cancer. Chem Biol Interact.

[21] Cobb MH, Goldsmith EJ (1995). How MAP kinases are regulated. J Biol Chem.

[22] Wagner EF, Nebreda AR (2009). Signal integration by JNK and p38 MAPK pathways in cancer development. Nat Rev Cancer.

[23] Lee HG, Minematsu H, Kim KO (2011). Actin and ERK1/2-CEBPβ signaling mediates phagocytosis-induced innate immune response of osteoprogenitor cells. Biomaterials.

[24] McGlynn LM, Kirkegaard T, Edwards J (2009). Ras/Raf-1/MAPK pathway mediates response to tamoxifen but not chemotherapy in breast cancer patients. Clin Cancer Res.

[25] Atmaca A, Pauligk C, Steinmetz K (2011). Prognostic impact of phosphorylated mitogen-activated protein kinase expression in patients with metastatic gastric cancer. Oncology.

[26] Wasserman WW, Fahl WE (1997). Functional antioxidant responsive elements. Proc Natl Acad Sci USA.

[27] Li Y, Jaiswal AK (1992). Regulation of human NAD(P)H: quinone oxidoreductase gene. Role of AP1 binding site contained within human antioxidant response element. J Biol Chem.

[28] Rushmore TH, Pickett CB (1990). Transcriptional regulation of the rat glutathione S-transferase Ya subunit gene. Characterization of a xenobiotic-responsive element controlling inducible expression by phenolic antioxidants. J Biol Chem.

[29] Prestera T, Holtzclaw WD, Zhang Y, Talalay P (1993). Chemical and molecular regulation of enzymes that detoxify carcinogens. Proc Natl Acad Sci USA.

